# Plasma Lipids Profile in the Prediction of Non-Alcoholic Steatohepatitis in Adults: A Case-Control Study

**DOI:** 10.3390/ijms241612717

**Published:** 2023-08-12

**Authors:** Georgios Kalopitas, Thomai Mouskeftara, Theodoros Liapikos, Konstantinos Arvanitakis, Aristeidis Ioannidis, Konstantinos Malandris, Eleni Theocharidou, Michail Chourdakis, Emmanouil Sinakos, Helen Gika, Georgios Germanidis

**Affiliations:** 1 Division of Gastroenterology and Hepatology, 1st Department of Internal Medicine, AHEPA University Hospital, School of Medicine, Faculty of Health Sciences, Aristotle University of Thessaloniki, 54636 Thessaloniki, Greece; gekalopi@auth.gr (G.K.); kostarvanit@gmail.com (K.A.); 2 Basic and Translational Research Unit, Special Unit for Biomedical Research and Education, School of Medicine, Faculty of Health Sciences, Aristotle University of Thessaloniki, 54636 Thessaloniki, Greece; 3 Laboratory of Hygiene, Social and Preventive Medicine and Medical Statistics, School of Medicine, Faculty of Health Sciences, Aristotle University of Thessaloniki, 54636 Thessaloniki, Greece; mhourd@gapps.auth.gr; 4 Laboratory of Forensic Medicine & Toxicology, Department of Medicine, Aristotle University of Thessaloniki, 54124 Thessaloniki, Greece; mousthom@auth.gr (T.M.); gkikae@auth.gr (H.G.); 5 Biomic AUTh, Center for Interdisciplinary Research and Innovation (CIRI-AUTH), Balkan Center B1.4, 10th km Thessaloniki-Thermi Rd., 57001 Thessaloniki, Greece; tliapikos@chem.auth.gr; 6 Laboratory of Analytical Chemistry, Department of Chemistry, Aristotle University of Thessaloniki, 54124 Thessaloniki, Greece; 7 1st Propedeutic Department of Surgery, AHEPA University Hospital of Thessaloniki, Aristotle University of Thessaloniki, 54636 Thessaloniki, Greece; ariioann@yahoo.gr; 8 2nd Department of Internal Medicine, Hippokration General Hospital, Aristotle University of Thessaloniki, 54642 Thessaloniki, Greece; kostas_malandris@yahoo.gr (K.M.); eltheocharidou@hotmail.com (E.T.); 9 4th Medical Department, Hippokratio Hospital, Aristotle University of Thessaloniki, 54642 Thessaloniki, Greece; esinakos@auth.gr

**Keywords:** plasma lipids, acylcarnitines, fatty acids, ceramides, non-alcoholic steatohepatitis, non-alcoholic fatty liver disease, diagnostic markers, non-invasive diagnosis

## Abstract

Patients with non-alcoholic steatohepatitis (NASH) show significantly faster progress in the stages of fibrosis compared to those with non-alcoholic fatty liver (NAFL) disease. The non-invasive diagnosis of NASH remains an unmet clinical need. Preliminary data have shown that sphingolipids, especially ceramides, fatty acids, and other lipid classes may be related to the presence of NASH and the histological activity of the disease. The aim of our study was to assess the association of certain plasma lipid classes, such as fatty acids, acylcarnitines, and ceramides, with the histopathological findings in patients with NASH. The study included three groups: patients with NASH (N = 12), NAFL (N = 10), and healthy [non non-alcoholic fatty liver disease (NAFLD)] controls (N = 15). Plasma samples were collected after 12 h of fasting, and targeted analyses for fatty acids, acylcarnitines, and ceramides were performed. Baseline clinical and demographic characteristics were collected. There was no significant difference in baseline characteristics across the three groups or between NAFL and NASH patients. Patients with NASH had increased levels of several fatty acids, including, among others, fatty acid (FA) 14:0, FA 15:0, FA 18:0, FA 18:3n3, as well as Cer(d18:1/16:0), compared to NAFL patients and healthy controls. No significant difference was found between NAFL patients and healthy controls. In conclusion, patients with NASH exhibited a distinctive plasma lipid profile that can differentiate them from NAFL patients and non-NAFLD populations. More data from larger cohorts are needed to validate these findings and examine possible implications for diagnostic and management strategies of the disease.

## 1. Introduction

Non-alcoholic fatty liver disease (NAFLD) is considered a modern pandemic affecting more than 25% of the general population worldwide [1]. Its prevalence is constantly rising in parallel with the prevalence of οbesity and metabolic syndrome (MetS). NAFLD is considered the hepatic component of the latter. The spectrum of the disease includes non-alcoholic fatty liver (NAFL), which is characterized by steatosis with or without mild inflammation, and the more progressive non-alcoholic steatohepatitis (NASH), which is characterized by the coexistence of steatosis, inflammation, and hepatocellular ballooning [1,2] with or without fibrosis.

Regarding the prognosis of the disease, the most important prognostic indicator is the presence of advanced fibrosis. Steatohepatitis seems to be an important driver for the progression of more advanced stages of fibrosis. It has been demonstrated that patients with NAFL progress by one stage of fibrosis on average every 14 years, while patients with NASH show faster disease progression of one fibrosis stage every 7 years. It is evident that the early diagnosis of NASH is clinically important, as it can prompt early interventions in the disease course, leading to a subsequent reduction in morbidity and mortality in a significant part of the general population [3,4]. Even though there are reliable non-invasive modalities to assess liver fibrosis, there are currently no non-invasive modalities to assess for the presence and grade of NASH. Transient elastography and several non-invasive serological tests (NITs), such as FIB-4 and NAFLD fibrosis score, can rule out or rule in clinically significant liver fibrosis with high sensitivity and specificity [5,6]. On the other hand, the diagnosis of NASH can only be made histologically with a liver biopsy. The limitations of liver biopsy include increased costs (due to the need for hospitalization and follow-up) and a small risk of life-threatening complications. It is evident that there is an increasing need for a reliable non-invasive diagnostic biomarker for NASH.

It has been recently proposed that plasma lipids may have a role in the non-invasive diagnosis of NASH [7,8]. Recent studies found notable alterations in lipid composition in liver biopsies of patients with NAFLD (such as phospholipids, fatty acids, and sphingolipids), indicating that disturbances in the metabolism of specific lipid species could be a potential contributor to the pathogenesis of NAFLD/NASH. Puri et al. [7] assessed hepatic lipid composition in 27 liver biopsies and found that NAFLD is associated with alterations in the hepatic lipid profile. Moreover, changes in the circulating plasma lipid species might also be associated with disease progression. Apostolopoulou et al. [9] conducted a prospective study including bariatric patients. They analysed liver, serum, and adipose tissue ceramides and found that sphingolipid levels were higher in patients with insulin resistance and NASH, and their levels correlated with the degree of hepatic inflammation and oxidative stress. A small number of further lipidomic studies have been performed in recent years [10,11,12,13,14,15,16] with important methodological limitations. Most of these studies included morbidly obese/bariatric patients and often heterogeneous populations. The majority of these studies examined only hepatic and not plasma lipids, which potentially could be used for the non-invasive diagnosis of NASH. Finally, the studies that examined both plasma and hepatic lipids showed inconsistency between measured parameters.

The aim of our study was to investigate potential differences in plasma lipids in patients with NASH, NAFL, and healthy controls that may help differentiate NASH from NAFL and non-invasively assess the histological activity of the disease. To achieve this goal, we applied multitargeted analyses by liquid chromatography–mass spectrometry (LC–MS) and gas chromatography–mass spectrometry (GC–MS) for the quantification of plasma fatty acids, acylcarnitines, and ceramides. Moreover, state-of-the-art statistical machine learning methods were used for the analysis of the data.

## 2. Results

### 2.1. Characteristics of the Study Population

Our study included 37 participants: 15 healthy volunteers, 10 subjects with NAFL, and 12 subjects with NASH. The median age was 54 years, and 22 (59.4%) were male. There were no differences across the three groups in terms of age and genre. The median BMI of the total population was 29.3 kg/m^2^. Patients with NASH and NAFL had significantly higher BMIs compared to healthy controls, but the difference between the former two groups was not significant. MetS and its individual components (arterial hypertension, T2DM, increased WC, and dyslipidemia) were more common in NASH than in NAFL patients and were more common in NAFL patients than healthy controls. AST, HbA1c, and triglyceride values were higher in NASH compared to NAFL patients. Baseline characteristics and biochemical parameters are summarized in Table 1.

### 2.2. Serum Lipids Concentrations

The concentrations of individual lipid species were considered in the three groups, as well as lipid sums, ratios, and indexes that are indicative of de novo lipogenesis (DNL), which is highly related to NAFLD and NASH pathogenesis. As hepatic steatosis is consistently observed with an attenuation of the polyunsaturated to monounsaturated fatty acids ratio [17], the sum of monounsaturated fatty acids (MUFA), non-essential fatty acids (NEFA), saturated fatty acids (SFA), polyunsaturated fatty acids (PUFA), and the lipogenic index calculated as the ratio of palmitic acid (16:0) to the essential omega-6 linoleic acid (18:2), reflecting DNL rates, was assessed. In addition, desaturation and elongation indices were evaluated. The abbreviations of the plasma lipids determined, used in the tables and figures of this manuscript are presented in supplementary Table S1.

**Figure 1:**
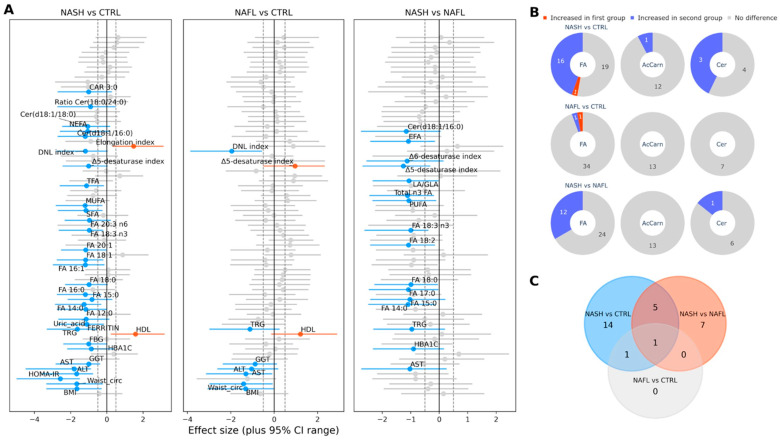
Effect size analysis of the differences among the study groups. (**A**) Effect size plots of all the parameters studied: The calculated effect size of each variable is depicted along with the corresponding 95% CI. Variables that had an effect size value beyond the +/− 0.5 limit (gray dashed vertical lines) and simultaneously present a statistically significant difference with a Mann–Whitney U test *p* value < 0.05 are colored red and blue, respectively. (**B**) Pie chart with a comparison of the number of plasma lipid species (total fatty acids (FA), acylcarnitines (AcCarn), and ceramides (Cer)) that showed significant changes among the 3 groups: For each pair of groups compared, we have a record of the category of the plasma lipids compared. The criteria for a significant change and the color code are the same as those in Figure 1A. (**C**) Venn diagram showing the number of common significantly differentiated lipid species in the paired comparisons of the three groups of subjects: The common lipids for the NASH vs. NAFLD and NASH vs. control pairs are FA 14:0, 15:0, 18:0, 18:3n3, Δ5-desaturase index, and Cer(d18:1/16:0) μmol/L.

Fatty acid (FA) composition was significantly different in NASH patients compared to NAFL patients. Specifically, fatty acids FA 14:0, FA 15:0, FA 17:0, FA 18:0, FA 18:2, and FA 18:3n3 were significantly elevated in the NASH group. The LA/GLA ratio, levels of essential fatty acids (EFA), total polyunsaturated fatty acids (PUFA), and total n3-fatty acids were also significantly increased. The NASH group had higher Δ5 and Δ6 desaturase indexes. Regarding ceramide levels, NASH patients had significantly higher levels of Cer(d18:1/16:0) in comparison to NAFL patients. Similarly, in NASH patients, fatty acids FA 12:0, FA 14:0, FA 15:0, FA 16:0, FA 16:1, FA 18:0, FA 18:3n3, FA 20:1, FA 20:3n6, and FA 18:1 were upregulated in comparison to healthy controls. Essential fatty acids (EFA), non-essential fatty acids (NEFA), total fatty acids (TFA), total saturated fatty acids (SFA), total monounsaturated fatty acids (MUFA), the Δ5-desaturase index, the de novo lipogenesis (DNL) index, and the elongation index were elevated in NASH patients compared to controls. Levels of Cer(d18:1/16:0), Cer(d18:1/18:0), and the Cer(d18:1/18:0)/Cer(d18:1/24:0) ratio were significantly different in the NASH group compared to healthy controls. NAFL patients had no significant differences in fatty acid levels compared to healthy controls, with the exception of the Δ5-desaturase index and DNL index values. No significant difference in acylcarnitines levels was found between the NASH group and the other two groups, with the exception of CAR 3:0, which differed significantly between NASH and the controls. The plasma lipids, where statistically significant changes were found, are shown in Table 2. The detailed plasma lipid analysis is presented in supplementary Table S2. Figure 1 presents the differences among the three study groups in an “effect size” analysis.

### 2.3. Subgroup Analysis Based on HOMA IR

A subgroup analysis was performed of the total study population based on the homeostasis model assessment of insulin resistance (HOMA-IR) values. The study population was further stratified into two groups, a low (<3) and a high HOMA-IR (>3) group. The “high HOMA-IR” group had a higher BMI (31.8 vs. 25.3 kg/m^2^) and WC. The presence of MetS and its individual components were more common in the same group. Patients with NASH had higher HOMA-IR values, while only 20% of normal controls had high HOMA-IR values. The “high HOMA-IR” group had increased levels of liver function tests (ALT, AST, GGT, ALP), ferritin, and uric acid. Characteristics of the two groups are presented in Table 3.

Serum lipid concentrations were elevated in high HOMA-IR compared to low HOMA-IR patients. Particularly, fatty acids FA 12:0, FA 14:0, FA 15:0, FA 16:0, FA 17:0, FA 18:0, FA 16:1, FA 18:1, FA 18:2, and FA 20:1 were significantly increased in the high HOMA-IR group. The LA/GLA and AA/EPA ratios, as well as SFA, MUFA, PUFA, and TFA, were significantly increased. The DNL and elongation index were higher in the HOMA-IR group. In the low HOMA-IR group, Cer(d18:1/18:0), the Cer(d18:1/18:0)/Cer(d18:1/24:0) ratio, CAR 3:0, and CAR 5:0 were lower compared to the high HOMA-IR group. Lipid profiles for the two groups are summarized in Table 4 and are presented in more detail in supplementary Table S3.

### 2.4. Correlation of Lipid Profiles with the Histologic Activity of NAFLD

We assessed correlations between clinical and biochemical characteristics and plasma lipid profiles, and NAS score and fibrosis score in NAFLD patients.

There was a positive correlation between the NAS score and transaminase, triglyceride, HOMA-IR, and uric acid levels. Among plasma lipid parameters, levels of several fatty acids, including FA 16:1, FA 16:0, FA 14:0, the elongation index, NEFA, MUFA, SFA, and FA 18:1, had a strong correlation with the NAS score. Regarding fibrosis stage, HOMA-IR, FBG, HBA1C, GGT, and FA 12:0 were strongly correlated with the fibrosis score. These data are presented in Table 5, and the detailed correlations are presented in supplementary Table S4.

### 2.5. Diagnostic Performance of Clinical, Biochemical, and Plasma Lipid Profiles in Predicting NASH

We calculated the area under the curve (AUC) to assess the ability of different parameters in discriminating NASH from NAFL/healthy controls. The index of Δ5-desaturase had an AUC of 0.854 (0.64–0.99), FA 18:3 n3 (ALA) had an AUC of 0.85 (0.69–0.99), and FA 14:0, FA 17:0, PUFA, EFA, and FA 18:2 had AUC values greater than 0.8. AST values had an AUC of 0.825 (0.63–0.99).

**Figure 2:**
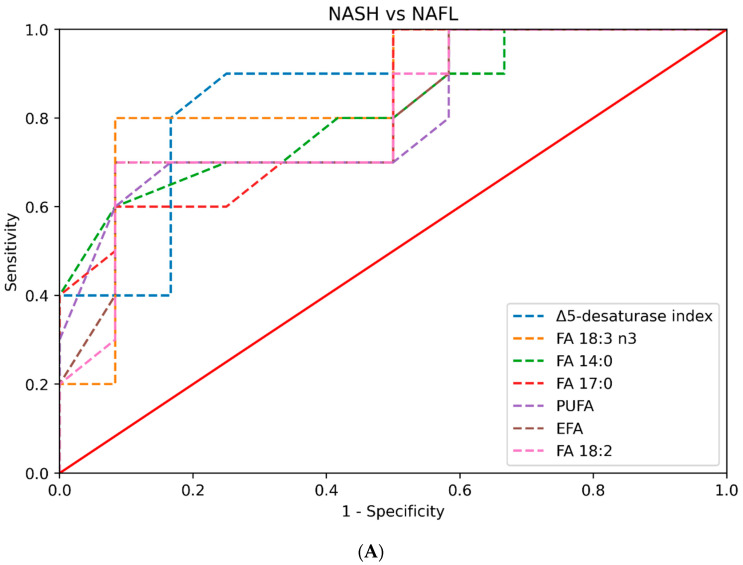

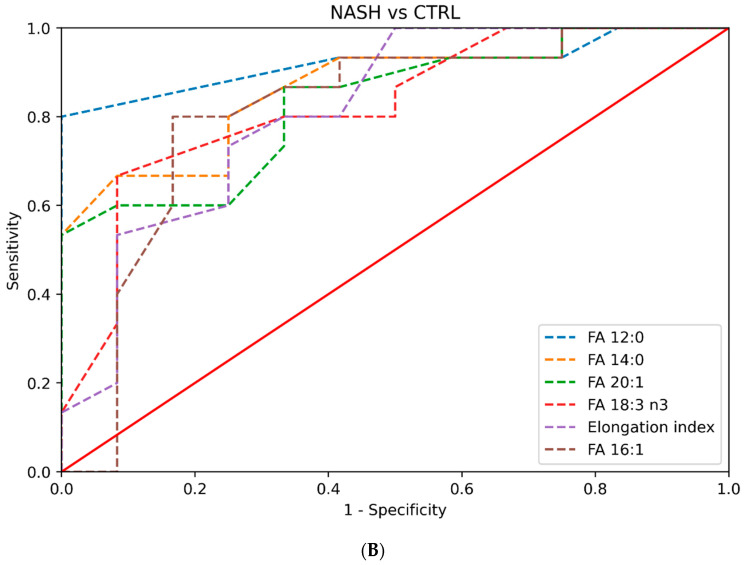
(**A**) Plot of the AUC values of the plasma lipid species in the prediction of NASH in comparison to NAFL (only AUC values greater than 0.8 are depicted). (**B**) Plot of the AUC values of the plasma lipid species in the prediction of NASH in comparison to healthy controls (only AUC values greater than 0.8 are depicted).

When we assessed the ability of plasma lipid parameters to discriminate between NASH and healthy controls, FA 12:0 had an AUC value of 0.92 (0.81–1); FA 14:0 had an AUC value of 0.87 (0.71–0.98); and FA 20:1, FA 18:3 n3, the elongation index, and FA 16:1 had AUC values greater than 0.8. The AUC values of the plasma lipids with an AUC > 0.8 for each pair of groups compared are presented in Table 6 and Figure 2. The AUC values for all the plasma lipids are presented in Supplementary Table S5.

## 3. Discussion

The non-invasive diagnosis of NASH remains a clinical challenge, as there is currently no available biomarker that can discriminate NASH from NAFL. The diagnosis of NASH can only be made histologically. However, the large number of NAFLD patients and the invasive nature of liver biopsy render this approach problematic. The aim of this study was to characterize lipid profiles (plasma fatty acids, acylcarnitines, and ceramides) in patients with NASH and NAFL and to investigate potential correlations with histological activity and fibrosis.

Patients were classified as having NASH or NAFL based on liver histology. Patients with NAFL and NASH had similar metabolic profiles, but had more metabolic risk factors compared to controls, as expected. A major finding of our study is that patients with NASH had a distinctive plasma lipid profile from patients with NAFL, although the two groups did not differ in terms of established metabolic risk factors. In addition, patients with NAFL and healthy controls had similar plasma lipid profiles, even though the former had significantly more metabolic risk factors.

Patients with NASH had significant differences in the values of several species of fatty acids, such as FA 12:0, FA 14:0, FA 15:0, FA 16:1, FA 17:0, FA 18:0, FA 18:3n3, FA 18:2, the LA/GLA ratio, EFA, TFA, SFA, total n3-FAs, and others, compared to NAFL patients and healthy controls. These results indicate that specific lipotoxic fatty acids, mainly saturated fatty acids, may play a role in the pathogenesis of NASH. The intrahepatic accumulation of saturated fatty acids probably induces oxidative stress and activates the inflammasome in the liver, resulting in hepatocyte injury and apoptosis [18]. Given the fact that the samples in our study were taken after an overnight fasting, it is unlikely that this finding is an effect of dietary intake, but it probably is a result of increased DNL and increased peripheral lipolysis (due to insulin resistance). The fact that the products of DNL are exclusively saturated fatty acids further supports this rationale. This trend was also demonstrated in a previous study carried out by Puri et al. [19], although this study had important methodological limitations, such as differences between the study groups. Similar findings were also observed in a study conducted by Walle et al. [16], which found increased serum concentrations of saturated fatty acids, mainly FA 14:0, FA16:0, and FA 18:0, in patients with NASH compared to patients with NAFL. Another study that was conducted by Luukonen et al. [12] showed increased levels of saturated fatty acids in the livers of adults with NASH. Similar results have also been found in recent animal studies [20]. The existence of a genetic predisposition probably contributes to these findings. This was seen in a previous study conducted by our group, which concluded that the *rs738409* polymorphism of the *PNPLA3* gene affects the composition of the blood fatty acids [21]. This polymorphism resulted in increased concentrations of blood saturated fatty acids such as palmitic, stearic, oleic, and linoleic acids, the values of which were found to be significantly elevated in NASH patients in our study. In contrast to previous lipidomic studies, the levels of PUFA in our study were increased [8,19].

Regarding ceramides, in our study, Cer(d18:1/16:0) levels were significantly increased in NASH patients compared to NAFL patients and healthy controls, and Cer(d18:1/18:0) levels and the Cer(d18:1/18:0)/Cer(d18:1/24:0) ratio were significantly higher in NASH patients compared to healthy controls. Increased concentrations of C 16:0 ceramide were also observed both in recent animal studies [22,23], which found that this specific ceramide’s concentrations were increased in obesity-related insulin resistance, and in a human study carried out by Luukonen et al. [12] in patients with NASH and insulin resistance. These findings indicate that the “de novo” ceramide synthesis pathway is probably upregulated in NASH. Ceramides are bioactive molecules that play an important role in many cellular functions and have been associated with insulin resistance, altered insulin signaling, as well as inflammatory and apoptotic processes [24]. These lipotoxic intermediates are considered important mediators in hepatocellular injury in NASH.

Another important finding of our study is the association between several fatty acid levels and histological activity, as assessed using the NAS score. In addition, several lipids, such as Δ5-desaturase index, FA 14:0, FA 17:0, FA 18:2, FA 18:3 n3, and PUFA, exhibit high accuracy in discriminating NASH from NAFL. This suggests that the plasma lipid profile might prove a useful tool in the non-invasive diagnosis of NASH. In contrast, the plasma lipid profile did not correlate with the fibrosis stage, except for fatty acid FA 12:0. However, safe conclusions could not be drawn due to the relatively small sample size of our study.

Several previous lipidomic/metabolomic studies attempted to further investigate the complex pathophysiology of NAFLD [7,8,9,10,11,12,13,14,15]. These studies yielded encouraging results regarding the different qualitative and quantitative hepatic lipid compositions in NAFLD patients in comparison to healthy subjects. However, most of these studies had several limitations. Most of these studies included morbidly obese patients undergoing bariatric surgery, which raises concerns as to the effect that extreme BMI levels might have on the circulating plasma lipidomic footprint [7,9,12,13,14,15]. Moreover, the absence of steatosis was confirmed histologically in some of the studies, whereas others relied on the combination of normal liver ultrasound scans and normal liver biochemistry. Ultrasonography has the limitation that it can detect hepatic steatosis when at least 20–30% of the liver parenchyma is affected [25]. In addition, performing a liver biopsy in a patient with a normal liver raises bioethical issues when there is an equivalent, safe, and accurate non-invasive way to assess for hepatic steatosis, such as MRI-PDFF, which was used in our study [26,27]. Moreover, most of the studies focused on hepatic lipid composition, which might reflect more precisely the alterations in lipid metabolism that occur in NAFLD/NASH, but liver tissue is a biological material that is not easily accessible in daily clinical practice. In the studies that examined both plasma and hepatic lipids, the results were inconsistent in plasma and liver tissue. Finally, discrepancies were found in the results between the different studies that examined plasma lipids, possibly because of the different methods used, the heterogeneity in the populations examined, and the study designs.

Our study had substantial strengths. Firstly, we included three well-characterized groups. NAFL and NASH were confirmed histologically, and the absence of steatosis was confirmed by means of MRI-PDFF. Participants were common NAFLD patients seen in hepatology clinics and not patients at extreme ends of the MetS, such as morbidly obese patients (median BMI of NAFL and NASH patients were 31.1 and 31.6 kg/m^2^, respectively, and 25.3 kg/m^2^ in the control group). Moreover, a subgroup analysis was performed according to the HOMA-IR, which added further to our understanding of the role of lipids in NAFLD [28]. State-of-the-art statistical methods were also used. Regarding the methodology followed in our study for the plasma lipid profiles, it should be noted that it was specifically developed in our lab with the aim to assess dysregulated lipid profiles in clinical samples and to ensure the accurate determination of the certain lipid species.

Our study has some limitations. The relatively small number of participants is the main limitation. Another limitation is that a targeted lipidomic analysis was conducted that included lipid species that are thought to contribute to NASH pathogenesis according to currently available data. An untargeted lipidomic analysis that could identify more (all detectable) lipid species that potentially contribute to the pathogenesis of the disease could provide a more comprehensive pattern. This is the next study to be performed in these samples, and results will be soon published to build on the present data.

In summary, the results of our study indicate that NASH patients exhibit a distinct plasma lipid profile that is distinctive from NAFL patients and non-NAFLD controls. This profile seems to correlate with histological activity. Based on these results, plasma lipids may provide a useful biomarker for the diagnosis of NASH. Our findings might have significant implications, facilitating earlier diagnosis and the personalized management of NASH, leading to improved patient outcomes and reduced disease burden. The results of our study require validation in larger cohorts.

## 4. Materials and Methods

### 4.1. Study Design

The current study was a case-control study that included three well characterized groups: patients with biopsy-proven NAFL, patients with biopsy-proven NASH, and healthy controls. All participants were enrolled in our study between June 2021 and June 2023 after providing written informed consent. The study was performed according to the principles of the Declaration of Helsinki. The study was approved by the Institutional Review Board and the Bioethics Board of Medical School of Aristotle University of Thessaloniki (protocol number 4.399/26/01/2021).

### 4.2. Study Cohort

Consecutive adult subjects with a recent (or suspected) diagnosis of NAFLD (within 6 months) that attended the hepatology outpatient clinic at AHEPA University Hospital (Thessaloniki, Greece) during the study period were screened for eligibility after providing written informed consent. Inclusion criteria were age > 18 years and a probable diagnosis of NAFLD based on the presence of hepatic steatosis on imaging studies (ultrasound scan, CT, or MRI scan), the presence of metabolic risk factors, and the exclusion of other causes for hepatic steatosis. Exclusion criteria were alcohol consumption > 20 gr/d in women and >30 gr/day in men. Patients with concomitant liver diseases (chronic viral hepatitis, autoimmune and cholestatic liver diseases, Wilson’s disease, hemochromatosis, a-1-antitrypsin deficiency, and drug-induced liver disease) were also excluded. Finally, subjects with underlying severe systemic diseases (such as cancer and end-stage liver, kidney, and heart disease) were excluded from our study.

Healthy controls were recruited from June 2021 to June 2023. In this group, the absence of hepatic steatosis was determined by normal liver biochemistry and a liver fat fraction < 5% on MRI-PDFF [26,27]. All healthy controls had a complete liver screen that was negative for chronic liver diseases.

Participants had clinical assessment, physical examination, and blood tests at baseline. The anthropometric and demographic parameters that were recorded were age, gender, weigh, height, body mass index (BMI), and waist circumference (WC). Baseline biochemistry parameters included: alanine aminotransferase (ALT), aspartate aminotransferase (AST), gamma-glutamyl transferase (GGt), alkaline phosphatase (ALP), billrubin, serum albumin, total cholesterol, high density lipoprotein (HDL), low density lipoprotein (LDL), triglycerides, fasting blood glucose (FBG), insulin, HbA1c, and ferritin.

Patients with NAFLD, who had indications according to current clinical practice guidelines [1,2], underwent ultrasound-guided percutaneous liver biopsy [1,2]. Liver biopsies were examined by an expert liver pathologist, who was blinded to patient characteristics. The definitive diagnosis of NAFLD was established by the presence of steatosis in at least 5% of the hepatocytes. The NAFLD activity score (NAS) and fibrosis score by the NASH Clinical Research Network (CRN) were used to grade inflammatory activity and stage fibrosis, respectively [29]. The NAS score consists of three components: steatosis (0–3), lobular inflammation (0–3), and hepatocellular ballooning (0–2), and it ranges from 0 to 8. The fibrosis score ranges from 0 (absence of fibrosis) to 4 (cirrhosis). Subjects with NAFLD were further divided into NAFL and NASH based on histological findings. Subjects with steatosis with no or mild inflammation were classified as NAFL. Subjects with at least 1 grade of each component of the NAS score were classified as NASH [30].

Blood samples were taken from all participants for plasma lipid analysis by three different methods (in subjects with NAFLD on the morning of the liver biopsy) after overnight fasting and a low-fat diet in the past 24 h. Blood samples were centrifugated, and plasma was separated and immediately stored at −80 °C.

### 4.3. Acylcarnitines, Ceramides and Fatty Acids Analyses

For the analysis of acylcarnitines and ceramides, two LC-MS methods developed in our lab were applied. For acylcarnitines, an hydrophilic interaction liquid chromatography tandem mass spectrometry (HILIC–MS/MS) method was applied quantifying 13 acylcarnitine analogues, namely Acetyl-L-Carnitine (CAR 2:0); Propionyl-L-Carnitine (CAR 3:0); Butyryl-L-Carnitine (CAR 4:0); Valeryl-L-Carnitine (CAR 5:0); Hexanoyl-L-Carnitine (CAR 6:); Octanoyl-L-Carnitine (CAR 8:0); Decanoyl-L-Carnitine (CAR 10:0); Lauroyl-L-Carnitine (CAR 12:0); Myristoyl-L-Carnitine (CAR 14:0); Palmitoyl-L-Carnitine (CAR 16:0); Stearoyl-L-Carnitine (CAR 18:0); Oleoyl-L-carnitine (CAR 18:1); and Linoleoyl-L-Carnitine (CAR 18:2) [31,32].

For ceramides, the reverse phase LC–MS/MS method, quantifying four species, namely N-Palmitoyl-D-erythro-sphingosine (Cer d18:1/16:0); N-stearoyl-D-erythro-sphingosine (Cer d18:1/18:0); N-lignoceroyl-D-erythro-sphingosine (Cer d18:1/24:0); and N-nervonoyl-D-erythro-sphingosine (Cer d18:1/24:1), was applied [33]. Analysis was performed on an Acquity UPLC System (Waters Corporation, Milford, CT, USA) coupled on a XEVO TQD Mass Spectrometer (Waters Corporation, Milford, CT, USA). Data acquisition and analysis were performed by Waters MassLynx version 4.1 and TargetLynx (Waters, Milford, MA 01757, USA).

Total fatty acid analysis was performed on an Agilent Technologies 8860 GC, combined with a 5977 MSD (Agilent Technologies, Santa Clara, CA, USA). Fatty acid methyl esters were separated on an Agilent 100 m HP-88 column (0.25 μm, i.d. of 0.25 μm). Data were processed by MassHunter Workstation (Version 10.0) software. Fatty acids were extracted from serum samples using a modified Folch protocol followed by methanolysis/methylation under acidic conditions, as described in our previous study [21]. In total, 20 fatty acid methyl esters were quantified, namely Lauric (FA 12:0), Myristic (FA 14:0), Pentadecanoic (FA 15:0), Palmitic (FA 16:0), Heptadecanoic (FA 17:0), Stearic (FA 18:0), Arachidic (FA 20:0), Behenic (FA 22:0), Lignoceric (FA 24:0), Palmitoleic (FA 16:1), *cis*-9-Oleic (FA 18:1), *cis*-11-Eicosenoic (FA 20:1), Nervonic (FA 24:1), Linoleic (FA 18:2), Gamma Linolenic (FA 18:3n6), Alpha Linolenic (FA 18:3n3), Dihomogamma Linolenic (FA 20:3n6), Arachidonic (FA 20:4n6), *cis*-5,8,11,14,17 Eicosapentaenoic (FA 20:5n3), *cis*-4,7,10,13,16,19 Docosahexaenoic (FA 22:6n3) acids.

### 4.4. Data and Statistical Analysis

The Python (v. 3.10.6) programming language was used for statistical computations and the visualization of the results on a Linux OS based PC. The continuous variables are expressed as medians with interquartile range (IQR, 25th–75th percentile), while the categorical variables are expressed as counts and percentages for each variable’s category. The differences between the variables’ categories were examined using the Mann–Whitney U test for the continuous variables and the Chi-square (χ^2^) test for the categorical variables. Receiver operating characteristic (ROC) curves were plotted, and the area under the ROC (AUROC) and the corresponding *p* value were calculated to determine the diagnostic accuracy of the lipids to differentiate NASH from NAFL and CTRL. The sensitivity and the specificity for cutoffs were also calculated for specific lipids. Two-tailed *p* values < 0.05 were considered to indicate statistically significant differences in variables between groups. In addition, the effect size offering valuable information about the practical significance of a finding, independent of sample size, was calculated, along with its 95% confidence interval range, as a metric to quantify the magnitude of the observed differences in the study [34]. Cohen’s d was used as an effect size measure, which calculated the standardized mean difference between two groups by dividing the difference in means by the pooled standard deviation. Effect size values greater than 0.5 and 0.8 indicated medium and large effects, respectively. Confidence intervals were calculated using the bootstrapping method, which involves the repeated random resampling of the original dataset with the replacement [35]. Correlations between lipids and histological features were assessed by Pearson’s correlation.

### 4.5. Predetermined Subgroup Analysis

A subgroup analysis was performed based on the values of the homeostasis model assessment of insulin resistance (HOMA-IR) to examine patients that were insulin-resistant and, therefore, at a higher risk of suffering from NASH. Due to the lack of a globally accepted cut-off value of HOMA-IR that can differentiate insulin-resistant from insulin-sensitive subjects, a cut-off value of 3 was used. The total study population was divided into two groups: individuals with HOMA-IR values > 3 were classified into the “High HOMA-IR” group, while individuals with HOMA-IR values < 3 were classified into the “Low HOMA-IR” group.

## 5. Conclusions

In conclusion, our study demonstrated that subjects with NASH have a distinct plasma lipid profile compared to subjects with NAFL and healthy controls. This finding has multiple clinical, diagnostic, and therapeutic implications; however, due to the relatively small sample size of our study, larger-scale studies are needed to validate our results.

## Figures and Tables

**Table 1:** Baseline characteristics and biochemical parameters of the study population.

Variables	Total Population	Control Group	NAFL	NASH	NAFL vs. Control ^$^	NASH vs. Control ^$^	NASH vs. NAFL ^$^
N (Male)	37 (22)	15 (8)	10 (6)	12 (8)	0.7422	0.4835	0.7462
Age (years)	54.0 (46.0–60.0)	53.0 (41.5–55.0)	57.0 (53.2–60.0)	57.5 (45.8–65.5)	0.1261	0.3046	0.9211
BMI (kg/m^2^)	29.3 (24.9–31.8)	25.3 (24.0–28.7)	31.1 (26.6–33.0)	31.6 (28.6–35.2)	0.0158 *	0.0023 **	0.4483
Waist circumference (cm)	100 (95.0–112)	95.0 (83.5–98.0)	106 (97.0–118)	111 (100–120)	0.0105 *	0.0030 **	0.6678
HOMA-IR	3.50 (1.90–6.20)	1.90 (1.05–2.55)	3.30 (1.92–8.00)	6.10 (4.80–8.88)	0.0958	<0.0001 **	0.1378
T2DM	13 (35.1%)	3 (20%)	3 (30%)	7 (58.3%)	0.5663	0.0404 *	0.1839
Arterial hypertension	12 (32.4%)	3 (20%)	4 (40%)	5 (41.7%)	0.2752	0.2205	0.9369
Metabolic syndrome	20 (54.1%)	3 (20%)	6 (60%)	11 (91.7%)	0.0412 *	0.0002 **	0.0776
NAS	2 (0–3)	0	2.5 (2–3)	5 (4–6)	-	<0.0001 **	<0.0001 **
ALT (U/L)	28.0 (22.0–51.0)	20.0 (15.5–26.0)	35.5 (23.2–50.5)	48.5 (39.0–92.5)	0.0211 *	0.0001 **	0.1134
AST (U/L)	27.0 (20.0–39.0)	20.0 (18.0–23.5)	27.0 (23.8–31.0)	43.5 (35.8–52.2)	0.0224 *	0.0001 **	0.0111 *
GGT (U/L)	23.0 (13.0–54.0)	13.0 (11.0–20.0)	25.0 (18.5–56.2)	52.5 (35.0–93.5)	0.0169 *	0.0021 **	0.1378
ALP (U/L)	72.0 (55.0–93.0)	58.0 (53.5–89.0)	77.5 (62.8–96.8)	86.5 (64.8–94.2)	0.1741	0.2040	0.9212
Platelets(×10^3^) (K/μL)	226 (182–274)	217 (206–255)	253 (187–314)	215 (175–251)	0.4708	0.4208	0.3068
HBA1C (%)	5.50 (5.20–6.00)	5.40 (5.10–5.70)	5.50 (5.22–5.75)	6.15 (5.72–7.02)	0.5969	0.0335 *	0.0407 *
FBG (mg/dl)	96.0 (88.0–110)	89.0 (84.5–96.5)	94.5 (85.0–111)	109 (94.8–134)	0.4370	0.0167 *	0.2611
Insulin (μIU/mL)	12.3 (7.40–25.8)	9.50 (4.40–11.2)	14.4 (7.45–31.6)	25.4 (21.9–27.8)	0.1492	<0.0001 **	0.2913
Total cholesterol (mg/dl)	185 (147–202)	177 (155–200)	192 (125–215)	192 (168–200)	0.8243	0.6605	0.8174
LDL (mg/dl)	101 (77.0–126)	99.0 (76.0–128)	114 (60.5–126)	96.0 (81.0–120)	0.7602	0.9611	1.0000
HDL (mg/dl)	50.0 (42.0–60.0)	56.0 (53.5–64.0)	45.5 (32.2–54.8)	43.0 (40.8–49.2)	0.0324 *	0.0024 **	0.7659
Triglycerides (mg/dl)	116 (85.0–178)	84.0 (60.0–100)	128 (95.2–150)	192 (144–283)	0.0116 *	0.0002 **	0.0478 *
Ferritin (ng/mL)	149 (79.7–249)	97.0 (65.2–160)	136 (84.4–293)	200 (140–564)	0.1341	0.0104 *	0.3068
Uric acid (mg/dl)	4.90 (4.30–5.80)	4.80 (4.00–5.10)	4.85 (4.70–5.92)	5.60 (4.40–6.75)	0.2549	0.0427 *	0.4092
Albumin (gr/dl)	4.58 (4.38–4.70)	4.60 (4.36–4.70)	4.57 (4.51–4.68)	4.50 (4.36–4.62)	0.8225	0.5571	0.5521

Continuous variables are presented as median (25th–75th percentile). Categorical variables are presented as counts and percentage for each variable’s category. In the categorical variables, subjects are presented that have the disease. **^$^**: In the comparison between the groups, the *p* values were calculated using the Mann–Whitney U test for the continuous variables and the Chi2 (χ^2^) test for the categorical variables. *: *p* value < 0.05, **: *p* value < 0.01, statistically significant.

**Table 2:** Plasma lipid levels in the three study groups.

	Study Groups				*p* Values ^$^		
	Total (N = 37)	Controls (n = 15)	NAFL (n = 10)	NASH (n = 12)	NAFL vs. Control ^$^	NASH vs. Control ^$^	NASH vs. NAFL ^$^
Fatty Acids, Summary of Fatty Acids and Ratios							
FA 12:0 μmol/L	73.4 (71.4–75.3)	71.7 (71.2–72.6)	72.6 (70.7–75.0)	75.2 (73.8–85.9)	0.4540	0.0002 **	0.0602
FA 14:0 μmol/L	156 (147–171)	148 (143–160)	149 (143–165)	176 (162–209)	0.8029	0.0014 **	0.0134 *
FA 15:0 μmol/L	24.0 (22.7–30.5)	23.5 (22.2–28.8)	23.1 (21.0–26.0)	26.9 (25.1–34.9)	0.6774	0.0429 *	0.0272 *
FA 16:0 μmol/L	1716 (1531–2240)	1537 (1492–1786)	1694 (1610–1836)	2281 (1687–2944)	0.3899	0.0137 *	0.0927
FA 17:0 μmol/L	28.5 (25.4–35.8)	28.5 (25.2–34.2)	25.8 (24.6–32.3)	32.9 (28.0–41.6)	0.5603	0.0673	0.0134 *
FA 18:0 μmol/L	650 (577–727)	650 (570–684)	601 (565–674)	753 (601–804)	0.7184	0.0429 *	0.0229 *
FA 16:1 μmol/L	145 (129–184)	134 (121–144)	144 (128–174)	228 (154–334)	0.4212	0.0058 **	0.0518
FA 18:1 μmol/L	1333 (1134–1715)	1244 (1016–1394)	1316 (1149–1512)	1714 (1305–2750)	0.4212	0.0137 *	0.1062
FA 20:1 μmol/L	9.42 (8.82–9.99)	9.06 (8.41–9.55)	9.03 (8.66–10.3)	9.91 (9.41–11.3)	0.5603	0.0058 **	0.0806
FA 18:2 μmol/L	1522 (1374–1775)	1519 (1412–1672)	1326 (1277–1628)	1699 (1513–2380)	0.0907	0.1021	0.0161 *
FA 18:3 n3 μmol/L	66.0 (64.9–69.9)	65.3 (63.7–67.1)	65.3 (64.9–65.6)	70.8 (66.9–81.4)	0.7603	0.0043 **	0.0062 **
FA 20:3 n6 μmol/L	206 (196–218)	199 (193–207)	205 (196–215)	214 (205–232)	0.5982	0.0381 *	0.1985
SFA mmol/L	2.80 (2.63–3.49)	2.64 (2.54–2.94)	2.72 (2.64–3.03)	3.55 (2.76–4.28)	0.4540	0.0104 *	0.0602
MUFA mmol/L	1.53 (1.37–1.99)	1.46 (1.24–1.62)	1.53 (1.39–1.74)	2.08 (1.50–3.23)	0.4212	0.0137 *	0.1062
PUFA mmol/L	2.70 (2.45–2.94)	2.70 (2.49–2.84)	2.44 (2.38–2.80)	2.87 (2.64–3.58)	0.2555	0.1128	0.0134 *
Total n3 FA mmol/L	0.32 (0.31–0.34)	0.32 (0.30–0.33)	0.31 (0.29–0.32)	0.35 (0.31–0.36)	0.4540	0.1500	0.0378 *
TFA mmol/L	6.85 (6.50–8.52)	6.85 (6.14–7.40)	6.73 (6.44–7.40)	8.76 (6.82–11.09)	0.9337	0.0264 *	0.0518
LA/GLA	20.0 (18.0–22.0)	19.6 (18.9–20.7)	17.8 (15.6–20.0)	22.0 (19.9–26.2)	0.0806	0.1021	0.0321 *
Δ5-desaturase index	2.95 (2.63–3.31)	2.88 (2.79–3.12)	2.61 (2.40–2.90)	3.35 (3.07–4.33)	0.0429 *	0.0264 *	0.0062 **
Δ6-desaturase index	10.5 (9.74–12.0)	10.5 (9.93–11.5)	9.56 (8.64–10.9)	11.2 (10.4–14.7)	0.0631	0.2319	0.0378 *
DNL index	1.13 (1.04–1.27)	1.06 (1.02–1.09)	1.26 (1.18–1.34)	1.16 (1.11–1.33)	0.0017 **	0.0264 *	0.6209
Elongation index	0.36 (0.34–0.38)	0.37 (0.36–0.40)	0.36 (0.32–0.38)	0.33 (0.28–0.36)	0.1741	0.0078 **	0.2766
EFA μmol/L	1696 (1506–1921)	1661 (1548–1816)	1464 (1423–1778)	1853 (1681–2542)	0.0907	0.0923	0.0192 *
NEFA μmol/L	3833 (3470–4935)	3620 (3202–4005)	3831 (3484–4005)	5029 (3620–6912)	0.5235	0.0180 *	0.1213
Ceramides and Ceramides Ratio							
Cer(d18:1/16:0) μmol/L	0.59 (0.44–0.65)	0.49 (0.42–0.63)	0.56 (0.38–0.62)	0.70 (0.54–0.76)	0.8461	0.0232 *	0.0378 *
Cer(d18:1/18:0) μmol/L	0.24 (0.18–0.31)	0.18 (0.13–0.29)	0.23 (0.18–0.30)	0.29 (0.25–0.37)	0.2917	0.0264 *	0.1211
Ratio Cer(18:0/24:0)	0.02 (0.02–0.03)	0.02 (0.01–0.02)	0.02 (0.02–0.03)	0.03 (0.02–0.030)	0.1924	0.0205 *	0.3390
Acylcarnitines							
CAR 3:0 μmol/L	0.57 (0.51–0.72)	0.53 (0.50–0.60)	0.54 (0.42–0.70)	0.66 (0.58–0.77)	0.8461	0.0157 *	0.1379

Values of the plasma lipids are presented as median (25th–75th percentile). **^$^**: In the comparison between the groups, the *p* values were calculated using the Mann–Whitney U test. *: *p* value < 0.05, **: *p* value < 0.01: statistically significant.

**Table 3:** Baseline characteristics and biochemical parameters according to HOMA-IR.

Variables	Total Population	Low HOMA-IR	High HOMA-IR	Low vs. High HOMA-IR (*p* Value) ^$^
N (Male)	37 (22)	18 (10)	19 (12)	0.6378
Healthy controls	15	12 (80%)	3 (20%)	
NAFL	10	5 (50%)	5 (50%)	
NASH	12	0	12 (100%)	
Age (years)	54.0 (46.0–60.0)	53.0 (44.0–58.2)	56.0 (52.0–62.5)	0.1318
BMI (kg/m^2^)	29.3 (24.9–31.8)	25.3 (24.2–29.0)	31.8 (29.4–34.4)	0.0003 **
Waist circumference (cm)	100 (95.0–112)	95.0 (85.0–99.0)	112 (102–119)	0.0002 **
HOMA-IR	3.50 (1.90–6.20)	1.80 (0.92–2.28)	6.20 (4.65–8.95)	<0.0001 **
T2DM	13 (35.1%)	4 (22.2%)	9 (47.4%)	0.1093
Arterial hypertension	12 (32.4%)	3 (16.7%)	9 (47.4%	0.0462 *
Metabolic syndrome	20 (54.1%)	2 (11.1%)	18 (94.7%)	<0.0001 **
ALT (U/L)	28.0 (22.0–51.0)	21.5 (16.0–27.5)	44.0 (29.0–80.5)	0.0002 **
AST (U/L)	27.0 (20.0–39.0)	20.0 (18.0–26.8)	36.0 (27.0–49.5)	0.0002 **
GGT (U/L)	23.0 (13.0–54.0)	15.0 (11.2–20.8)	51.0 (28.0–94.0)	0.0003 **
ALP (U/L)	72.0 (55.0–93.0)	58.5 (53.2–69.8)	93.0 (74.5–108)	0.0013 **
Platelets (×10^3^) (K/μL)	226 (182–274)	223 (208–280)	245 (177–265)	0.6054
HBA1C (%)	5.50 (5.20–6.00)	5.40 (5.12–5.60)	5.90 (5.35–6.60)	0.0571
FBG (mg/dL)	96.0 (88.0–110)	91.0 (87.2–97.8)	102 (91.0–126)	0.1136
Insulin (μIU/mL)	12.3 (9.60–22.5)	7.20 (4.40–9.90)	25.8 (20.3–28.3)	<0.0001 **
Total cholesterol (mg/dL)	185 (164–199)	172 (149–199)	194 (176–202)	0.5333
LDL (mg/dL)	101 (77.0–126)	96.5 (75.5–125)	110 (80.0–124)	0.6815
HDL (mg/dL)	50.0 (42.0–60.0)	56.0 (52.5–64.0)	42.0 (37.5–49.5)	0.0008 **
Triglycerides (mg/dL)	116 (85.0–178)	84.5 (62.8–111)	178 (129–262)	<0.0001 **
Ferritin (ng/mL)	149 (79.7–249)	99.0 (66.3–169)	181 (105–502)	0.0193 *
Uric acid (mg/dL)	4.90 (4.30–5.80)	4.75 (3.92–5.00)	5.60 (4.65–6.60)	0.0075 **
Albumin (gr/dL)	4.58 (4.38–4.70)	4.60 (4.43–4.70)	4.53 (4.34–4.65)	0.2926

Continuous variables are presented as median (25th–75th percentile). Categorical variables are presented as counts and percentage for each variable’s category. In the categorical variables, subjects that have the disease are presented. -**^$^**: In the comparison between the groups, the *p* values were calculated using the Mann–Whitney U test for the continuous variables and the Chi2 (χ^2^) test for the categorical variables. -*: *p* value < 0.05, **: *p* value < 0.01, statistically significant.

**Table 4:** Plasma lipid composition in high and low HOMA-IR groups.

	Total (N = 37)	Low HOMA-IR (n = 18)	High HOMA-IR (n = 19)	Low vs. High HOMA-IR ^$^
Fatty Acids, Summary of Fatty Acids, and Ratios				
FA 12:0 μmol/L	73.4 (71.5–75.3)	71.7 (71.2–72.7)	75.2 (73.7–80.4)	0.0010 **
FA 14:0 μmol/L	156 (147–171)	148 (143–156)	167 (156–192)	0.0030 **
FA 15:0 μmol/L	24.0 (22.7–30.5)	23.0 (21.4–24.0)	26.6 (23.9–33.2)	0.0217 *
FA 16:0 μmol/L	1716 (1531–2240)	1612 (1514–1718)	2095 (1684–2664)	0.0085 **
FA 17:0 μmol/L	28.5 (25.4–35.8)	25.9 (24.8–32.5)	33.8 (27.5–38.6)	0.0373 *
FA 18:0 μmol/L	650 (577–727)	600 (565–672)	719 (601–780)	0.0200 *
FA 16:1 μmol/L	145 (129–184)	131 (120–144)	179 (144–274)	0.0033 **
FA 18:1 μmol/L	1333 (1134–1715)	1272 (1097–1384)	1582 (1193–2232)	0.0275 *
FA 20:1 μmol/L	9.42 (8.82–9.99)	9.01 (8.49–9.56)	9.90 (9.19–10.82)	0.0144 *
FA 18:2 μmol/L	1522 (1374–1775)	1430 (1328–1618)	1666 (1473–2140)	0.0465 *
SFA mmol/L	2.80 (2.63–3.49)	2.64 (2.57–2.85)	3.36 (2.76–3.98)	0.0065 **
MUFA mmol/L	1.53 (1.37–1.99)	1.48 (1.29–1.60)	1.94 (1.44–2.57)	0.0235 *
PUFA mmol/L	2.70 (2.45–2.94)	2.56 (2.41–2.77)	2.86 (2.55–3.34)	0.0298 *
TFA mmol/L	6.85 (6.50–8.52)	6.70 (6.39–7.22)	8.42 (6.81–9.48)	0.0157 *
LA/GLA	20.0 (18.0–22.0)	18.9 (17.6–20.2)	20.6 (19.4–24.4)	0.0465 *
AA/EPA	5.86 (5.63–6.27)	5.72 (5.33–5.94)	6.26 (5.77–6.42)	0.0157 *
DNL index	1.13 (1.04–1.27)	1.07 (1.02–1.23)	1.17 (1.09–1.32)	0.0402 *
Elongation index	0.36 (0.34–0.38)	0.37 (0.35–0.39)	0.34 (0.29–0.37)	0.0157 *
EFA μmol/L	1696 (1506–1921)	1571 (1467–1770)	1814 (1615–2301)	0.0465 *
NEFA μmol/L	3833 (3470–4935)	3577 (3339–3964)	4935 (3588–5903)	0.0144 *
Ceramides and Ceramides Ratio				
Cer (d18:1/18:0) μmol/L	0.24 (0.18–0.31)	0.18 (0.13–0.23)	0.29 (0.25–0.35)	0.0038 **
Ratio Cer (18:0/24:0)	0.02 (0.02–0.03)	0.02 (0.01–0.02)	0.03 (0.02–0.03)	0.0040 **
Acylcarnitines				
CAR 3:0 μmol/L	0.57 (0.51–0.72)	0.53 (0.50–0.60)	0.62 (0.56–0.78)	0.0465 *
CAR 5:0 μmol/L	0.06 (0.05–0.07)	0.05 (0.05–0.07)	0.07 (0.06–0.09)	0.0235 *

Values of the plasma lipids are presented as the median (25th–75th percentile). **^$^**: In the comparison between the groups, the *p* values were calculated using the Mann–Whitney U test. *: *p* value < 0.05, **: *p* value < 0.01, statistically significant.

**Table 5:** Significant correlations between baseline characteristics and plasma lipids values and the histological activity of NAFLD.

Variables	NAS Score *	Fibrosis Score *
ALT	0.6929	0.2742
HOMA-IR	0.6532	0.6312
AST	0.6384	0.3078
TRG	0.6075	0.3859
FA 16:1	0.5265	0.325
Elongation index	−0.5236	−0.4253
NEFA	0.5196	0.3184
MUFA	0.5194	0.3385
FA 16:0	0.5185	0.305
Uric acid	0.5162	0.0502
FA 14:0	0.5105	0.3631
SFA	0.5101	0.3015
FA 18:1	0.5065	0.3503
FBG	0.3981	0.5594
HBA1C	0.3171	0.5154
GGT	0.3668	0.5084
FA 12:0	0.3998	0.5277

* Correlations are presented with Pearson’s correlation coefficient (r), which ranges from −1 to +1.

**Table 6:** AUC values of the plasma lipids with an AUC > 0.8 for each pair of groups compared.

NASH vs. Control Comparison			Cut-Offs			
Lipids	AUROC (95% CI)	*p* Value	Cut-Off Point	AUROC (95% CI)	Sensitivity	Specificity
FA 12:0	0.92 (0.80–1)	<0.001 **	73.3	0.90 (0.79–1)	0.800	1
FA 14:0	0.87 (0.72–0.97)	<0.001 **	152	0.79 (0.63–0.93)	0.667	0.917
FA 20:1	0.83 (0.67–0.96)	<0.001 **	9.72	0.77 (0.60–0.93)	0.867	0.667
FA 18:3 n3	0.83 (0.66–0.97)	<0.001 **	66.1	0.79 (0.64–0.92)	0.667	0.917
Elongation index	0.81 (0.63–0.96)	0.132	0.324	0.75 (0.61–0.88)	1	0.500
FA 16:1	0.81 (0.61–0.96)	<0.001 **	145	0.82 (0.67–0.96)	0.800	0.833
**NASH vs. NAFL Comparison**			**Cut-Offs**			
**Lipids**	**AUROC (95% CI)**	***p*** **Value**	**Cut-Off Point**	**AUROC (95% CI)**	**Sensitivity**	**Specificity**
Δ5-desaturase index	0.85 (0.66–1)	<0.001 **	3.08	0.82 (0.64–0.96)	0.900	0.750
FA 18:3 n3	0.85 (0.67–1)	<0.001 **	66.1	0.86 (0.66–1)	0.800	0.917
FA 14:0	0.82 (0.59–0.96)	<0.001 **	152	0.76 (0.55–0.94)	0.600	0.917
FA 17:0	0.81 (0.61–0.94)	<0.001 **	26.5	0.76 (0.59–0.93)	0.600	0.917
PUFA	0.80 (0.57–0.97)	<0.001 **	2.52	0.77 (0.58–0.95)	0.700	0.833
EFA	0.80 (0.57–0.96)	<0.001 **	1.503	0.81 (0.62–0.96)	0.700	0.917
FA 18:2	0.80 (0.57–0.96)	0.074	1.382	0.81 (0.65–0.96)	0.700	0.917

The table contains (for the selected lipids and for each pair of groups compared): (a) The AUC values (95% CI) as well as the corresponding *p* value. (b) A defined cut-off point for each lipid, which is a threshold below from which the samples are classified in one category and above from this to the other. For these specific cut-offs, the AUC values (95% CI), as well as the sensitivity and specificity values, were recalculated. **: *p* value < 0.01, statistically significant.

## Data Availability

Data sharing not applicable.

## Supplementary Materials

The supporting information can be downloaded at: https://www.mdpi.com/article/10.3390/ijms241612717/s1.

## Abbreviations

ALA	Alfa-linoleic acid
ALP	Alkaline phosphatase
ALT	Alanine aminotransferase
AST	Aspartate aminotransferase
AUC	Area under the curve
BMI	Body mass index
CRN	Clinical Research Network
CT	Computed tomography
CVD	Cardiovascular disease
DGLA	Dihomo-γ-linolenic acid
FA	Fatty acid
FBG	Fasting blood glucose
GC–MS	Gas chromatography tandem mass spectrometry
GGT	Gamma-glutamyl transferase
HDL	High-density lipoprotein
HILIC-MS/MS	Hydrophilic interaction liquid chromatography tandem mass spectrometry
HOMA-IR	Homeostasis model assessment of insulin resistance
LA	Linoleic acid
LC–MS	Liquid chromatography–mass spectrometry
LDL	Low-density lipoprotein
MetS	Metabolic syndrome
MS	Mass spectrometry
NAFL	Non-alcoholic fatty liver
NAFLD	Non-alcoholic fatty liver disease
NAS	NAFLD activity score
NASH	Non-alcoholic steatohepatitis
NITs	Non-invasive tests
RPLC–MS/MS	Reversed phase liquid chromatography tandem mass spectrometry
T2DM	Type 2 diabetes mellitus
WC	Waist circumference
