# Supplementary material for: Plasma Lipids Profile in the Prediction of Non-Alcoholic Steatohepatitis in Adults: A Case-Control Study
Source: Int J Mol Sci. 2023 Aug 12;24(16):12717. doi: 10.3390/ijms241612717 (PMC10454013; doi:10.3390/ijms241612717)
Supplement: Supplementary file 1 [file ijms-24-12717-s001.zip › ijms-2557934-supplementary.pdf]

Supplementary material

Supplementary Table S1. Abbreviations of the plasma lipids calculated

Plasma lipid Name	Abbreviation	Category
C12:0 µmol/L	FA 12:0	Fatty acids
C14:0 µmol/L	FA 14:0	Fatty acids
C15:0 µmol/L	FA 15:0	Fatty acids
C16:0 µmol/L	FA 16:0	Fatty acids
C17:0 µmol/L	FA 17:0	Fatty acids
C18:0 µmol/L	FA 18:0	Fatty acids
C20:0 µmol/L	FA 20:0	Fatty acids
C22:0 µmol/L	FA 22:0	Fatty acids
C24:0 µmol/L	FA 24:0	Fatty acids
C16:1 µmol/L	FA 16:1	Fatty acids
C18:1 µmol/L	FA 18:1	Fatty acids
C20:1 µmol/L	FA 20:1	Fatty acids
C24:1 µmol/L	FA 24:1	Fatty acids
C18:2 (Linoleic acid=LA) µmol/L	FA 18:2	Fatty acids
C18:3 n3 (alfa linoleic acid, ALA) µmol/L	FA 18:3 n3	Fatty acids
C18:3 n6 (Gamma linoleic acid, GLA) µmol/L	FA 18:3 n6	Fatty acids
C20:3 n6 (Dihomo-γ-linolenic acid, DGLA)) µmol/L	FA 20:3 n6	Fatty acids
C20:4 n6 (Arachidonic acid, AA) µmol/L	FA 20:4 n6	Fatty acids
C20:5 n3 (Eicosapentaenoic acid, EPA) µmol/L	FA 20:5 n3	Fatty acids
C22:6 n3 (Docosahexadecaenoic acid, DHA) µmol/L	FA 22:6 n3	Fatty acids
Total saturated fatty acids mmol/L	SFA	Sum of fatty acids

Total monounsaturated fatty acids mmol/L	MUFA	Sum of fatty acids
Total polyunusaturated fatty acids mmol/L	PUFA	Sum of fatty acids
Total n3 fatty acids mmol/L	Total n3 FA	Sum of fatty acids
Total n6 fatty acids mmol/L	Total n6 FA	Sum of fatty acids
Total fatty acids mmol/L	TFA	Sum of fatty acids
LA/GLA	LA/GLA	Fatty acids ratio
EPA/DGLA	EPA/DGLA	Fatty acids ratio
AA/EPA	AA/EPA	Fatty acids ratio
D5-desaturase index (C18:2/C20:4)	Δ5-desaturase index	Fatty acids ratio
D6-desaturase index (C18:2/C18:3)	Δ6-desaturase index	Fatty acids ratio
D9-desaturase index (C16:1/C16:0)	Δ9-desaturase index	Fatty acids ratio
DNL index (C16:0/C18:2)	DNL index	Fatty acids ratio
Elongation index (C18:0/C16:0)	Elongation index	Fatty acids ratio
Essential fatty acids (C18:2+C18:3) µmol/L	EFA	Sum of fatty acids
Non-essential fatty acids (C16:0+C16:1+C18:0+C18:1) µmol/L	NEFA	Sum of fatty acids
Cer(d18:1/16:0) µmol/L	Cer(d18:1/16:0)	Ceramides
Cer(d18:1/18:0) µmol/L	Cer(d18:1/18:0)	Ceramides
Cer(d18:1/24:0) µmol/L	Cer(d18:1/24:0)	Ceramides
Cer(d18:1/24:1) µmol/L	Cer(d18:1/24:1)	Ceramides
Ratio Cer(d18:1/16:0)/ Cer(d18:1/24:0)	Ratio Cer(16:0/24:0)	Ceramides ratio
Ratio Cer(d18:1/18:0)/ Cer(d18:1/24:0)	Ratio Cer(18:0/24:0)	Ceramides ratio
Ratio Cer(d18:1/24:1)/ Cer(d18:1/24:0)	Ratio Cer(18:1/24:0)	Ceramides ratio
Acetylcarnitine C2 µmol/L	CAR 2:0	Acylcarnitines
Propionylcarnitine C3 µmol/L	CAR 3:0	Acylcarnitines
Butyrylcarnitine C4 µmol/L	CAR 4:0	Acylcarnitines
Valerylcarnitine C5 µmol/L	CAR 5:0	Acylcarnitines
Hexanoylcarnitine C6 µmol/L	CAR 6:0	Acylcarnitines

Octanoylcarnitine C8 µmol/L	CAR 8:0	Acylcarnitines
Decanoylcarnitine C10 µmol/L	CAR 10:0	Acylcarnitines
Lauroylcarnitine C12 µmol/L	CAR 12:0	Acylcarnitines
Myristoylcarnitine C14 µmol/L	CAR 14:0	Acylcarnitines
Palmitoylcarnitine C16 µmol/L	CAR 16:0	Acylcarnitines
Linoleoylcarnitine C18:2 µmol/L	CAR 18:2	Acylcarnitines
Oleoylcarnitine C18:1 µmol/L	CAR 18:1	Acylcarnitines
Stearoylcarnitine C18 µmol/L	CAR 18:0	Acylcarnitines

Supplementary Table S2. Detailed plasma lipid composition and comparison between the 3 study groups

	Study groups						
	Total	***Controls***	***NAFL***	***NASH***	***NAFL vs Control$***	***NASH vs Control$***	***NASH vs NAFL$***
N	**37**	**15**	**10**	**12**			
Fatty acids and analogues							
FA 12:0	73.3 (71.4-75.2)	71.7 (71.1-72.5)	72.5 (70.6-74.9)	75.2 (73.8-85.9)	0.4540	0.0002**	0.0602
FA 14:0	156 (146-170)	148 (142-159)	149 (142-164)	176 (161-209)	0.8029	0.0014**	0.0134*
FA 15:0	23.9 (22.7-30.4)	23.5 (22.1-28.7)	23.1 (21.0-25.9)	26.9 (25.1-34.9)	0.6774	0.0429*	0.0272*
FA 16:0	1715 (1530-2240)	1536 (1492-1785)	1694 (1609-1836)	2280 (1687-2943)	0.3899	0.0137*	0.0927
FA 17:0	28.5 (25.4-35.8)	28.5 (25.1-34.1)	25.7 (24.6-32.3)	32.9 (28.0-41.5)	0.5603	0.0673	0.0134*
FA 18:0	650 (577-727)	650 (570-683)	600 (564-673)	753 (601-804)	0.7184	0.0429*	0.0229*
FA 20:0	56.5 (55.7-57.5)	56.7 (55.8-57.3)	56.3 (55.4-56.9)	56.6 (56.2-58.3)	0.56	0.51	0.1764
FA 22:0	84.0 (82.1-85.8)	84.6 (82.2-86.4)	84.2 (82.1-84.8)	83.6 (82.5-86.8)	0.4881	1	0.575

FA 24:0	50.4 (49.7-51.2)	50.5 (49.8-51.3)	50.3 (49.4-50.8)	50.2 (49.7-51.3)	0.4887	0.981	0.454
FA 16:1	144 (129-183)	133 (120-143)	143 (128-173)	227 (153-333)	0.4212	0.0058**	0.0518
FA 18:1	1332 (1134-1715)	1243 (1015-1393)	1315 (1149-1512)	1714 (1304-2750)	0.4212	0.0137*	0.1062
FA 20:1	9.42 (8.82-9.99)	9.06 (8.41-9.55)	9.03 (8.66-10.3)	9.91 (9.41-11.2)	0.5603	0.0058**	0.0806
FA 24:1	71.4 (69.8-75.9)	75.7 (70.3-78.7)	71.2 (69.9-73.5)	70.8 (69.1-72.9)	0.1416	0.102	0.8175
FA 20:5 n3	89.2 (84.9-93.8)	91.1 (84.6-96.7)	87.4 (84.6-89.9)	91.5 (86.3-98.7)	0.1572	0.643	0.1213
FA 22:6 n3	160 (148-172)	160 (148-172)	159 (145-163)	173 (154-189)	0.4212	0.2319	0.1062
FA 18:2	1521 (1374-1774)	1518 (1412-1671)	1326 (1276-1628)	1698 (1513-2379)	0.0907	0.1021	0.0161*
FA 18:3 n3	65.9 (64.9-69.9)	65.2 (63.7-67.1)	65.2 (64.9-65.5)	70.8 (66.9-81.4)	0.7603	0.0043**	0.0062**
FA 18:3 n6	79.8 (73.8-84.1)	77.6 (74.1-81.9)	78.2 (74.4-82.2)	86.3 (73.8-92.3)	0.9337	0.1796	0.1765
FA 20:3 n6	205 (196-217)	199 (192-207)	205 (195-214)	214 (205-231)	0.5982	0.0381*	0.1985
FA 20:4 n6	528 (502-552)	528 (501-550)	527 (507-549)	535 (500-557)	0.9337	0.9029	0.9212
SFA	2.80 (2.63-3.49)	2.64 (2.54-2.94)	2.72 (2.64-3.03)	3.55 (2.76-4.28)	0.4540	0.0104*	0.0602
MUFA	1.53 (1.37-1.99)	1.46 (1.24-1.62)	1.53 (1.39-1.74)	2.08 (1.5-3.23)	0.4212	0.0137*	0.1062
PUFA	2.70 (2.45-2.94)	2.7 (2.49-2.84)	2.44 (2.38-2.8)	2.87 (2.64-3.58)	0.2555	0.1128	0.0134*
Total n3 FA	0.32 (0.31-0.34)	0.32 (0.3-0.33)	0.31 (0.29-0.32)	0.35 (0.31-0.36)	0.4540	0.1500	0.0378*
Total n6 FA	0.81 (0.78-0.86)	0.81 (0.77-0.84)	0.81 (0.79-0.85)	0.84 (0.78-0.9)	0.8029	0.2723	0.3734
TFA	6.85 (6.5-8.52)	6.85 (6.14-7.40)	6.73 (6.44-7.4)	8.76 (6.82-11.09)	0.9337	0.0264*	0.0518
LA/GLA	20.0 (17.9-21.9)	19.6 (18.8-20.7)	17.8 (15.6-20.0)	22.0 (19.9-26.2)	0.0806	0.1021	0.0321*
EPA/DGLA	0.44 (0.41-0.47)	0.45 (0.43-0.5)	0.42 (0.41-0.44)	0.41 (0.39-0.48)	0.1017	0.15	1

AA/EPA	5.86 (5.63-6.27)	5.74 (5.24-6.18)	6.15 (5.84-6.39)	5.79 (5.62-6.17)	0.0806	0.7511	0.1765
Δ5-desaturase index	2.95 (2.63-3.31)	2.88 (2.79-3.12)	2.61 (2.4-2.9)	3.35 (3.07-4.33)	0.0429*	0.0264*	0.0062**
Δ6-desaturase index	10.4 (9.74-11.9)	10.4 (9.93-11.4)	9.56 (8.64-10.8)	11.1 (10.4-14.6)	0.0631	0.2319	0.0378*
Δ9-desaturase index	0.08 (0.08-0.1)	0.08 (0.07-0.09)	0.09 (0.08-0.1)	0.1 (0.08-0.12)	0.1741	0.0832	0.1765
DNL index	1.13 (1.04-1.27)	1.06 (1.02-1.09)	1.26 (1.18-1.34)	1.16 (1.11-1.33)	0.0017**	0.0264*	0.6209
Elongation index	0.36 (0.34-0.38)	0.37 (0.36-0.4)	0.36 (0.32-0.38)	0.33 (0.28-0.36)	0.1741	0.0078**	0.2766
EFA	1695 (1506-1921)	1661 (1548-1816)	1463 (1422-1778)	1852 (1680-2542)	0.0907	0.0923	0.0192*
NEFA	3832 (3469-4935)	3620 (3202-4004)	3830 (3484-4005)	5028 (3620-6911)	0.5235	0.0180*	0.1213
Ceramides							
Cer(d18:1/16:0)	0.59 (0.44-0.65)	0.49 (0.42-0.63)	0.56 (0.38-0.62)	0.7 (0.54-0.76)	0.8461	0.0232*	0.0378*
Cer(d18:1/18:0)	0.24 (0.18-0.31)	0.18 (0.13-0.29)	0.23 (0.18-0.3)	0.29 (0.25-0.37)	0.2917	0.0264*	0.1211
Cer(d18:1/24:0)	10.7 (8.55-12.9)	10.2 (7.75-12.9)	10.0 (8.87-11.8)	11.7 (9.75-13.0)	0.7392	0.4067	0.2622
Cer(d18:1/24:1)	3.49 (2.65-4.47)	3.05 (2.67-4.5)	3.43 (2.65-3.67)	3.94 (3.04-4.63)	0.8897	0.2829	0.2225
Ratio Cer (16:0/24:0)	0.05 (0.04-0.06)	0.05 (0.04-0.05)	0.05 (0.04-0.06)	0.06 (0.05-0.07)	0.2917	0.0264	0.1211
Ratio Cer (18:0/24:0)	0.02 (0.02-0.03)	0.02 (0.01-0.02)	0.02 (0.02-0.03)	0.03 (0.02-0.03)	0.1924	0.0205*	0.3390
Ratio Cer (24:1/24:0)	0.35 (0.28-0.41)	0.32 (0.24-0.42)	0.35 (0.34-0.37)	0.33 (0.3-0.42)	0.8897	0.2830	0.2225
Acylcarnitines							
CAR 2:0	12.6 (8.31-15.1)	9.79 (8.25-16.5)	13.6 (8.92-16.1)	12.6 (10.5-14.8)	0.4881	0.9805	0.5310

CAR 4:0	0.09 (0.07-0.12)	0.07 (0.06-0.11)	0.08 (0.07-0.12)	0.11 (0.09-0.13)	0.5982	0.0603	0.2225
CAR 3:0	0.57 (0.51-0.72)	0.53 (0.5-0.6)	0.54 (0.42-0.7)	0.66 (0.58-0.77)	0.8461	0.0157*	0.1379
CAR 5:0	0.06 (0.05-0.07)	0.05 (0.05-0.07)	0.06 (0.05-0.07)	0.07 (0.06-0.1)	0.4373	0.0570	0.3390
CAR 6:0	0.06 (0.04-0.08)	0.06 (0.04-0.07)	0.07 (0.05-0.1)	0.07 (0.04-0.09)	0.5982	0.6430	1.0000
CAR 8:0	0.15 (0.1-0.19)	0.17 (0.13-0.19)	0.11 (0.09-0.17)	0.16 (0.08-0.23)	0.668	0.668	0.668
CAR 10:0	0.26 (0.14-0.33)	0.28 (0.21-0.33)	0.17 (0.14-0.26)	0.27 (0.12-0.37)	0.869	0.869	0.869
CAR 12:0	0.04 (0.03-0.06)	0.04 (0.03-0.06)	0.03 (0.03-0.05)	0.05 (0.02-0.08)	0.575	0.575	0.575
CAR 14:0	0.04 (0.03-0.05)	0.04 (0.03-0.04)	0.04 (0.03-0.04)	0.04 (0.03-0.05)	0.621	0.621	0.621
CAR 16:0	0.1 (0.09-0.14)	0.1 (0.09-0.12)	0.09 (0.08-0.13)	0.1 (0.09-0.14)	0.531	0.531	0.531
CAR 18:2	0.18 (0.14-0.26)	0.2 (0.16-0.27)	0.16 (0.12-0.2)	0.17 (0.14-0.26)	1.000	1.000	1.000
CAR 18:1	0.18 (0.14-0.24)	0.21 (0.15-0.27)	0.17 (0.15-0.24)	0.18 (0.13-0.21)	0.668	0.668	0.668
CAR 18:0	0.03 (0.02-0.03)	0.03 (0.02-0.04)	0.02 (0.02-0.03)	0.02 (0.02-0.03)	0.767	0.767	0.767

- Values of the plasma lipids are presented as median (25th-75th percentile)

-$: In the comparison between the groups the *P* values were calculated using the Mann Whitney U test

- *: p value <0.05. **: p value <0.01.: statistically significant

Supplementary table S3. Detailed plasma lipid composition and comparison between high and low HOMA-IR groups

	Total	***Low HOMA-IR***	***High HOMA-IR***	***Low vs High HOMA-IR $***
N	***37***	***18***	***19***	
Fatty acids and analogues				
FA 12:0	73.3 (71.4-75.2)	71.6 (71.1-72.6)	75.1 (73.6-80.4)	0.0010**
FA 14:0	156 (146-170)	148 (142-156)	166 (156-191)	0.0030**
FA 15:0	23.9 (22.7-30.4)	23.0 (21.4-24.0)	26.6 (23.9-33.1)	0.0217*
FA 16:0	1715 (1530-2240)	1612 (1514-1718)	2095 (1684-2664)	0.0085**
FA 17:0	28.5 (25.4-35.8)	25.9 (24.8-32.4)	33.7 (27.4-38.6)	0.0373*
FA 18:0	650 (577-727)	600 (564-672)	718 (600-779)	0.0200*
FA 20:0	56.5 (55.7-57.5)	56.4 (55.6-57.0)	56.6 (56.1-57.9)	0.3234
FA 22:0	84.0 (82.1-85.8)	84.1 (81.8-85.1)	84.0 (82.4-86.8)	0.3087
FA 24:0	50.4(49.7-51.2)	50.3 (49.6-50.9)	50.4 (49.8-51.4)	0.4566
FA 16:1	144 (129-183)	131 (119-144)	179 (143-274)	0.0033**
FA 18:1	1332 (1134-1715)	1271 (1097-1384)	1581 (1193-2231)	0.0275*
FA 20:1	9.42 (8.82-9.99)	9.01 (8.49-9.56)	9.9 (9.19-10.8)	0.0144*
FA 24:1	71.4 (69.8-75.9)	72.2 (69.7-76.3)	71.3 (70.1-74.8)	0.8673
FA 18:2	1521 (1374-1774)	1430 (1328-1617)	1665 (1472-2139)	0.0465*
FA 20:5 n3	89.2 (84.9-93.8)	90.7 (86.0-93.3)	86.9 (84.7-94.5)	0.3540
FA 22:6 n3	161 (148-178)	159 (147-163)	164 (153-181)	0.2300
FA 18:3 n3	65.9 (64.9-69.9)	65.3 (64.7-66.4)	68.9 (65.4-75.5)	0.0617
FA 18:3 n6	79.8 (73.8-84.1)	77.2 (74.9-81.7)	82.6 (73.7-87.9)	0.3700
FA 20:3 n6	205 (196-217)	199 (196-205)	213 (198-220)	0.0706
FA 20:4 n6	528 (502-552)	520 (497-539)	547 (507-575)	0.1325
SFA	2.8 (2.63-3.49)	2.64 (2.57-2.85)	3.36 (2.76-3.98)	0.0065**
MUFA	1.53 (1.37-1.99)	1.48 (1.29-1.6)	1.94 (1.44-2.57)	0.0235*
PUFA	2.7 (2.45-2.94)	2.56 (2.41-2.77)	2.86 (2.55-3.34)	0.0298*
TFA	6.85 (6.5-8.52)	6.7 (6.39-7.22)	8.42 (6.81-9.48)	0.0157*
LA/GLA	20.0 (17.9-21.9)	18.8 (17.6-20.2)	20.6 (19.4-24.3)	0.0465*
EPA/DGLA	0.44 (0.41-0.47)	0.44 (0.43-0.48)	0.42 (0.39-0.46)	0.0575
AA/EPA	5.86 (5.63-6.27)	5.72 (5.45-5.92)	6.26 (5.79-6.41)	0.0157*
Δ5-desaturase index	2.95 (2.63-3.31)	2.8 (2.63-3.04)	3.09 (2.78-3.88)	0.0575
Δ6-desaturase index	10.4 (9.74-11.9)	9.93 (9.56-10.83)	11.2 (10.31-13.3)	0.0617
Δ9-desaturase index	0.08 (0.08-0.1)	0.08 (0.08-0.09)	0.09 (0.08-0.1)	0.0660
DNL index	1.13 (1.04-1.27)	1.07 (1.02-1.23)	1.17 (1.09-1.32)	0.0402*
Elongation index	0.36 (0.34-0.38)	0.37 (0.35-0.39)	0.34 (0.29-0.37)	0.0157*
EFA	1695 (1506-1921)	1571 (1467-1769)	1813 (1614-2300)	0.0465*
NEFA	3832 (3469-4935)	3576 (3338-3963)	4935 (3588-5903)	0.0144*
Ceramides				
Cer(d18:1/16:0)	0.59 (0.44-0.65)	0.47 (0.42-0.61)	0.65 (0.51-0.71)	0.0575
Cer(d18:1/18:0)	0.24 (0.18-0.31)	0.18 (0.13-0.23)	0.29 (0.25-0.35)	0.0038**
Cer(d18:1/24:0)	10.7 (8.55-12.9)	10.1 (8.59-12.4)	10.8 (8.53-13.0)	0.5739
Cer(d18:1/24:1)	3.49 (2.65-4.47)	3.06 (2.56-3.67)	3.72 (2.91-4.64)	0.1212

Ratio Cer(16:0/24:0)	0.05 (0.04-0.06)	0.05 (0.04-0.05)	0.05 (0.05-0.06)	0.1862
Ratio Cer(18:0/24:0)	0.02 (0.02-0.03)	0.02 (0.01-0.02)	0.03 (0.02-0.03)	0.0040**
Ratio Cer(24:1/24:0)	0.35 (0.28-0.41)	0.31 (0.24-0.39)	0.35 (0.32-0.41)	0.2545
Acylcarnitines				
CAR 2:0	12.6 (8.31-15.1)	9.01 (8.3-16.3)	13.0 (10.9-14.8)	0.5535
CAR 3:0	0.57 (0.51-0.72)	0.53 (0.5-0.6)	0.62 (0.56-0.78)	0.0465*
CAR 4:0	*0.09 (0.07-0.12)*	0.08 (0.06-0.12)	0.1 (0.08-0.12)	0.1176
CAR 5:0	*0.06 (0.05-0.07)*	0.05 (0.05-0.07)	0.07 (0.06-0.09)	0.0235*
CAR 6:0	*0.06 (0.04-0.08)*	0.06 (0.04-0.08)	0.07 (0.05-0.09)	0.4846
CAR 8:0	*0.15 (0.1-0.19)*	0.16 (0.12-0.19)	0.13 (0.09-0.2)	0.7497
CAR 10:0	*0.26 (0.14-0.33)*	0.27 (0.2-0.33)	0.2 (0.14-0.32)	0.3234
CAR 12:0	*0.04 (0.03-0.06)*	0.04 (0.03-0.06)	0.04 (0.03-0.07)	0.9153
CAR 14:0	*0.04 (0.03-0.05)*	0.04 (0.03-0.04)	0.04 (0.03-0.05)	0.4474
CAR 16:0	*0.1 (0.09-0.14)*	0.1 (0.08-0.12)	0.1 (0.09-0.14)	1.0000
CAR 18:2	*0.18 (0.14-0.26)*	0.19 (0.13-0.24)	0.16 (0.15-0.26)	0.8197
CAR 18:1	*0.18 (0.14-0.24)*	0.19 (0.15-0.27)	0.17 (0.14-0.23)	0.3700
CAR 18:0	*0.03 (0.02-0.03)*	0.03 (0.02-0.03)	0.02 (0.02-0.03)	0.1621

- Values of the plasma lipids are presented as median (25th-75th percentile)
- $: In the comparison between the groups the *P* values were calculated using the Mann Whitney U test
- *: p value <0.05. **: p value <0.01. statistically significant

Supplementary table S4. Correlation of clinical, laboratory and plasma lipid parameters with NAS and Fibrosis score

	**NAS score***	**Fibrosis score***
**FA 16:1**	0,5265	0,2081
**FA 18:3 n 6**	0,3073	0,1755
**Cer(d18:1/18:0)**	0,4599	0,2606
**Cer(d18:1/24:0)**	0,2492	0,0533
**Cer(d18:1/24:1)**	0,2739	0,1172
**AA/EPA**	0,0655	0,0126
**CAR 2:0**	-0,0691	-0,088
**Age**	0,0823	0,3084
**ALB**	0,0348	-0,0676
**ALP**	0,1769	0,1999
**ALT**	0,6929	0,2742
**AST**	0,6384	0,3078
**BMI**	0,4711	0,4812
**CAR 4:0**	0,2421	-0,0081
**FA 12:0**	0,3998	0,5277
**FA 14:0**	0,5105	0,3631
**FA 15:0**	0,3483	0,369
**FA 16:0**	0,5185	0,305
**FA 17:0**	0,3421	0,3736
**FA 18:0**	0,4541	0,2377
**FA 18:3 n3**	0,3992	0,2518

**FA 20:0**	0,2227	0,1987
**FA 20:1n11**	0,4595	0,4309
**FA 20:3 n6**	0,419	0,0895
**FA 20:4 n6**	0,1377	0,0362
**FA 20:5 n3**	0,0162	0,0123
**FA 22:0**	0,0163	-0,0747
**FA 22:6 n3**	0,2985	0,1538
**FA 24:0**	0,0023	-0,1031
**FA 24:1**	-0,3467	-0,2893
**Cer(d18:1/16:0)**	0,3844	0,3283
**FA 18:1**	0,5065	0,3503
**FA 18:2**	0,412	0,2324
**Δ5-desaturase index**	0,4256	0,2595
**Δ6-desaturase index**	0,3588	0,2093
**Δ9-desaturase index**	0,282	0,0347
**CAR 10:0**	-0,0463	-0,0988
**DNL index**	0,4136	0,3352
**Elongation index**	-0,5236	-0,4253
**EPA/DGLA**	-0,3451	-0,0812
**EFA**	0,4138	0,2341
**FBG**	0,3981	0,5594
**FERRITIN**	0,3882	0,3801
**GGT**	0,3668	0,5084
**HBA1C**	0,3171	0,5154
**HDL**	-0,4441	-0,347
**CAR 6:0**	0,1165	0,1074
**HOMA-IR**	0,6532	0,6312
**LA/GLA**	0,3785	0,2226
**CAR 12:0**	0,1055	-0,0184
**LDL**	-0,0172	-0,119
**CAR 18:2**	-0,0967	-0,0258
**CAR 14:0**	0,1181	0,0639
**NEFA**	0,5196	0,3184
**CAR 8:0**	-0,0195	-0,0599
**CAR 18:1**	-0,1717	-0,0919
**CAR 16:0**	0,0576	0,0207
**PLT(x1000)**	-0,0922	-0,2431
**CAR 3:0**	0,3304	0,3101
**Ratio Cer(d18:1/16:0)/ Cer(d18:1/24:0)**	0,1679	0,3305
**Ratio Cer(d18:1/18:0)/ Cer(d18:1/24:0)**	0,3441	0,2664
**Ratio Cer(d18:1/24:1)/ Cer(d18:1/24:0)**	0,0298	0,0705
**CAR 18:0**	-0,1563	-0,0272
**TFA**	0,4972	0,2995
**MUFA**	0,5194	0,3385
**Total n3 FA**	0,3388	0,1924
**Total n6 FA**	0,2971	0,0798
**PUFA**	0,4153	0,2264

**SFA**	0,5101	0,3015
**TRG**	0,6075	0,3859
**Uric acid**	0,5162	0,0502
**CAR 5:0**	0,3476	0,2602
**WC**	0,4595	0,4318
* Correlations are presented with Pearson's correlation coefficient (r) which ranges from -1 to +1		

Supplementary Table S5. The AUC values for all the plasma lipids and each pair of groups compared

	NASH_CTRL		NASH_NAFL		NAFL_CTRL	
**Lipids**	**AUROC (95% CI)**	***P* value**	**AUROC (95% CI)**	***P* value**	**AUROC (95% CI)**	***P* value**
**AA/EPA**	0.53 (0.49-0.79)	0.735	0.67 (0.51-0.88)	0.139	0.72 (0.54-0.91)	0.045*
**CAR 10:0**	0.57 (0.51-0.82)	0.607	0.53 (0.49-0.82)	0.851	0.67 (0.51-0.88)	0.191
**CAR 12:0**	0.54 (0.51-0.80)	0.714	0.57 (0.50-0.80)	0.558	0.58 (0.50-0.78)	0.480
**CAR 14:0**	0.57 (0.50-0.79)	0.537	0.58 (0.50-0.80)	0.601	0.55 (0.50-0.79)	0.679
**CAR 16:0**	0.51 (0.50-0.76)	0.849	0.58 (0.51-0.83)	0.516	0.58 (0.50-0.85)	0.564
**CAR 18:0**	0.66 (0.51-0.86)	0.129	0.55 (0.50-0.80)	0.747	0.68 (0.52-0.92)	0.133
**CAR 18:1**	0.63 (0.52-0.85)	0.247	0.55 (0.50-0.79)	0.652	0.53 (0.51-0.80)	0.831
**CAR 18:2**	0.59 (0.51-0.80)	0.399	0.49 (0.50-0.83)	1.000	0.63 (0.51-0.90)	0.269
**CAR 2:0**	0.50 (0.50-0.80)	0.963	0.58 (0.50-0.81)	0.512	0.59 (0.50-0.83)	0.459
**CAR 3:0**	0.79 (0.61-0.94)	0.002**	0.69 (0.51-0.92)	0.126	0.54 (0.51-0.84)	0.846
**CAR 4:0**	0.72 (0.53-0.91)	0.031*	0.65 (0.51-0.88)	0.238	0.58 (0.50-0.80)	0.584
**CAR 5:0**	0.73 (0.54-0.89)	0.029*	0.61 (0.50-0.87)	0.339	0.59 (0.51-0.82)	0.431
**CAR 6:0**	0.55 (0.51-0.79)	0.640	0.50 (0.50-0.78)	1.000	0.56 (0.50-0.81)	0.601
**CAR 8:0**	0.52 (0.50-0.78)	0.928	0.55 (0.50-0.79)	0.648	0.66 (0.51-0.86)	0.238
**Cer(d18:1/16:0)**	0.76 (0.56-0.91)	0.006**	0.77 (0.54-0.94)	0.009**	0.54 (0.50-0.78)	0.828
**Cer(d18:1/18:0)**	0.76 (0.54-0.92)	0.007**	0.69 (0.52-0.91)	0.078	0.63 (0.51-0.84)	0.261
**Cer(d18:1/24:0)**	0.59 (0.51-0.79)	0.383	0.65 (0.50-0.88)	0.224	0.55 (0.50-0.78)	0.711
**Cer(d18:1/24:1)**	0.63 (0.50-0.82)	0.256	0.66 (0.51-0.89)	0.184	0.51 (0.50-0.75)	0.867
**DNL index**	0.76 (0.57-0.93)	0.010*	0.58 (0.50-0.83)	0.598	0.89 (0.71-1)	<0.001**
**EFA**	0.68 (0.51-0.89)	<0.001**	0.80 (0.57-0.96)	0.239	0.70 (0.52-0.95)	0.139
**EPA/DGLA**	0.67 (0.51-0.90)	0.066	0.51 (0.48-0.78)	0.002**	0.71 (0.52-0.89)	0.105
**Elongation index**	0.81 (0.63-0.96)	0.132	0.64 (0.51-0.87)	1.000	0.67 (0.51-0.86)	0.059
**FA 12:0**	0.92 (0.80-1)	<0.001**	0.75 (0.50-0.92)	0.035*	0.59 (0.49-0.83)	0.468
**FA 14:0**	0.87 (0.72-0.97)	<0.001**	0.82 (0.59-0.96)	0.001**	0.51 (0.50-0.79)	0.789
**FA 15:0**	0.72 (0.55-0.89)	0.016*	0.79 (0.57-0.95)	0.005**	0.55 (0.50-0.84)	0.660
**FA 16:0**	0.79 (0.58-0.96)	0.003**	0.72 (0.51-0.89)	0.049*	0.61 (0.51-0.85)	0.372
**FA 16:1**	0.81 (0.61-0.96)	0.001**	0.75 (0.55-0.92)	0.019*	0.59 (0.49-0.83)	0.408
**FA 17:0**	0.71 (0.52-0.88)	0.035*	0.81 (0.61-0.94)	0.001**	0.58 (0.50-0.82)	0.536
**FA 18:0**	0.74 (0.53-0.92)	0.022*	0.79 (0.58-0.94)	0.003**	0.55 (0.50-0.80)	0.694
**FA 18:1**	0.79 (0.61-0.93)	0.002**	0.71 (0.52-0.91)	0.065	0.60 (0.51-0.84)	0.396
**FA 18:2**	0.69 (0.51-0.89)	0.074	0.80 (0.57-0.96)	0.001**	0.70 (0.52-0.95)	0.105
**FA 18:3 n3**	0.83 (0.66-0.97)	<0.001**	0.85 (0.67-1)	<0.001**	0.53 (0.48-0.78)	0.737
**FA 18:3 n6**	0.65 (0.50-0.86)	0.190	0.68 (0.50-0.90)	0.157	0.51 (0.50-0.78)	0.912

**FA 20:0**	0.62 (0.46-0.83)	0.495	0.67 (0.49-0.86)	0.137	0.58 (0.48-0.79)	0.534
**FA 20:1**	0.83 (0.67-0.96)	<0.001**	0.73 (0.53-0.93)	0.061	0.57 (0.50-0.83)	0.550
**FA 20:3 n6**	0.74 (0.55-0.91)	0.020*	0.67 (0.51-0.88)	0.158	0.57 (0.49-0.80)	0.582
**FA 20:4 n6**	0.53 (0.49-0.76)	0.889	0.53 (0.48-0.78)	0.896	0.52 (0.49-0.81)	0.913
**FA 20:5 n3**	0.57 (0.49-0.80)	0.626	0.70 (0.53-0.91)	0.079	0.66 (0.52-0.86)	0.109
**FA 22:0**	0.49 (0.48-0.73)	1.000	0.60 (0.47-0.82)	0.558	0.59 (0.49-0.82)	0.456
**FA 22:6 n3**	0.64 (0.51-0.86)	0.208	0.72 (0.52-0.90)	0.062	0.6 (0.50-0.81)	0.403
**FA 24:0**	0.52 (0.50-0.72)	0.961	0.60 (0.48-0.83)	0.471	0.59 (0.48-0.81)	0.441
**FA 24:1**	0.68 (0.52-0.88)	0.067	0.53 (0.49-0.80)	0.793	0.67 (0.50-0.89)	0.095
**LA/GLA**	0.68 (0.52-0.91)	0.092	0.78 (0.56-0.95)	0.006**	0.71 (0.53-0.96)	0.079
**MUFA**	0.78 (0.61-0.93)	0.002**	0.70 (0.52-0.90)	0.062	0.61 (0.51-0.85)	0.393
**NEFA**	0.78 (0.60-0.93)	0.004**	0.70 (0.52-0.89)	0.078	0.58 (0.50-0.85)	0.507
**PUFA**	0.68 (0.51-0.89)	0.083	0.80 (0.57-0.97)	0.001**	0.65 (0.51-0.92)	0.277
**Ratio Cer(16:0/24:0)**	0.62 (0.51-0.84)	0.340	0.64 (0.51-0.87)	0.268	0.50 (0.50-0.82)	1.000
**Ratio Cer(18:0/24:0)**	0.77 (0.57-0.93)	0.004**	0.63 (0.50-0.85)	0.316	0.66 (0.50-0.87)	0.153
**Ratio Cer(18:1/24:0)**	0.54 (0.51-0.76)	0.697	0.50 (0.50-0.82)	1.000	0.52 (0.50-0.77)	0.868
**SFA**	0.79 (0.59-0.95)	0.001**	0.75 (0.54-0.91)	0.024*	0.59 (0.50-0.84)	0.436
**TFA**	0.75 (0.57-0.92)	0.008**	0.75 (0.56-0.92)	0.016*	0.51 (0.50-0.81)	0.914
**Total n3 FA**	0.65 (0.51-0.87)	0.128	0.77 (0.58-0.93)	0.010*	0.60 (0.50-0.80)	0.422
**Total n6 FA**	0.62 (0.50-0.83)	0.268	0.62 (0.50-0.86)	0.351	0.53 (0.50-0.80)	0.780
**Δ5-desaturase index**	0.75 (0.55-0.94)	0.015*	0.85 (0.66-1)	<0.001**	0.75 (0.56-0.97)	0.033*
**Δ6-desaturase index**	0.62 (0.50-0.86)	0.225	0.77 (0.56-0.95)	0.010*	0.73 (0.54-0.97)	0.069
**Δ9-desaturase index**	0.71 (0.52-0.91)	0.078	0.68 (0.51-0.89)	0.144	0.67 (0.51-0.85)	0.135

-The table includes for each pair of groups compared and for each lipid. the AUC value (95% CI). as well as the corresponding one P value of the difference from the dummy model

- *: p value <0.05. **: p value <0.01. statistically significant
